# Validation of a Rapid Method to Assess Habitual Beverage Intake Patterns

**DOI:** 10.3390/nu10010083

**Published:** 2018-01-13

**Authors:** Valisa E. Hedrick, Emily A. Myers, Jamie M. Zoellner, Kiyah J. Duffey, Brenda M. Davy

**Affiliations:** 1 Department of Human Nutrition, Foods, and Exercise, Virginia Tech, 295 West Campus Drive, Blacksburg, VA 24061, USA; eamyers@vt.edu (E.A.M.), bdavy@vt.edu (B.M.D.); 2 Department of Public Health Sciences, School of Public Health, University of Virginia, Cancer Center without Walls at the UVA Cancer Center, 16 East Main St, Christiansburg, VA 24073, USA; Jz9q@virginia.edu; 3 Department of Human Nutrition, Foods, and Exercise, Virginia Tech; Kiyah Duffey Consulting, Inc., 1807 Asher Lane, Blacksburg, VA 24060, USA; kiyah.duffey@gmail.com

**Keywords:** beverages, beverage quality, dietary patterns, dietary assessment, food frequency questionnaire

## Abstract

The Healthy Beverage Index (HBI) is an emerging approach to assess beverage pattern quality. HBI total scores range from 0 to 100, with higher scores indicating greater adherence to proposed beverage recommendations. However, assessing patterns is resource-intensive due to the need for extensive dietary data, typically 24-h dietary records or recalls. The BEVQ-15, a beverage intake questionnaire, may be used as an alternative method to rapidly measure HBI scores. The objective of this cross-sectional investigation is to assess the comparative validity of the HBI-Q, a method to rapidly assess HBI scores via the BEVQ-15, as compared to the traditional method of deriving HBI scores via dietary recalls/records. Between 2012 and 2016, a cross-sectional sample of adults in southwest Virginia completed three 24-h dietary recalls (30–60 min administration and analysis time per recall) and the BEVQ-15 (3–4 min administration time). HBI scores were generated by both methods, and compared via paired-samples *t*-tests, correlations, and Bland–Altman analysis. Among 404 adults (mean age = 40 years), total mean HBI scores were 63.7 from the HBI-Q and 67.3 from the recalls (mean difference = 3.6 out of 100; *r* = 0.63; both *p* ≤ 0.001). Agreement between the two methods for total HBI scores via Bland–Altman plots was 92%. Using the HBI-Q to rapidly assess HBI scores in adults will increase the utility of the HBI by decreasing the time and resources required, thus allowing researchers and practitioners to provide targeted feedback for improvement.

## 1. Introduction

As beverages currently contribute 19% of kcals to adults’ total daily energy intake in the United States (US) [1], beverage consumption provides a key target for improving a significant portion of one’s dietary intake. Beverage consumption influences health and weight status in numerous ways. For example, water consumption is associated with multiple health benefits, including a reduced risk of fatal coronary heart disease and gallstone formation [2], and increasing water consumption may be an important strategy for obesity prevention and treatment [3,4]. Sugar-sweetened beverage consumption, on the other hand, has been shown to promote weight gain and obesity [5,6], and reducing sugar-sweetened beverage intake may lower blood pressure and body weight, and reduce risk of diabetes and cardiovascular disease [7,8,9,10]. Other beverages have demonstrated positive associations with health: coffee and tea are associated with decreased incidence of cardiovascular disease and diabetes, and low-fat/fat-free milk intake is associated with bone density and can be an important source of calcium and Vitamin D [11,12].

There is evidence regarding interactions between single foods or nutrients and their impacts on health and disease outcomes. This has led to investigations of dietary patterns, which may better predict health or disease risk [13,14]. Specific to beverages, most of the attention has focused on sugar-sweetened beverage intake, with considerable controversy over health effects, taxation, and policy recommendations [9,15]. Yet, some have suggested that a “broader focus” is needed [16], and recent recommendations from the 2015 Dietary Guidelines for Americans suggest focusing on healthy dietary patterns, such as replacing sugar-sweetened beverages with water, or whole milk with skim milk [17]. Dietary pattern research has been specifically identified as a research gap by US Dietary Guidelines Committees [18]. Available research has demonstrated that healthier beverage patterns are associated with healthier overall dietary patterns [19]. Research identifying healthier overall dietary consumption patterns could be used to develop dietary recommendations that are more easily translated into optimal diets by the public [20], and to inform public policy.

Although national guidelines targeting improvements in beverage consumption patterns are currently being disseminated, there are no rapid methods of measuring the impact of programs and policies on actual beverage consumption or associated health outcomes. Presently, the Healthy Eating Index-2015, a measure of dietary quality which provides information about the extent to which diets conform to the 2015 Dietary Guidelines for Americans, is a commonly used tool to assess impact of new guidelines [21]. However, there are several important components of dietary intake not addressed by the Healthy Eating Index, including total fluid and water consumption and beverage energy intake, all of which may be important for optimal health [2,22,23]. Thus, the Healthy Beverage Index (HBI) was previously developed and validated to help overcome these limitations [24]. Similar to the Healthy Eating Index, the HBI serves as a measure of overall beverage quality, allowing for evaluation of the extent to which an individual’s beverage intake meets recommendations [24]. The HBI is a dietary quality tool that considers ten components of habitual beverage consumption to generate a beverage quality score [24]. However, like the Healthy Eating Index, the HBI requires the collection of extensive dietary intake data including total daily caloric intake, which can be burdensome to participants and to researchers (i.e., 3–4 day 24-h dietary recalls or records, with a 30–60 min administration and analysis time for each day).

To overcome this limitation and extend the utility of the HBI into clinical settings, clinical and community interventions, epidemiological studies, and population surveillance, a rapid measure of beverage consumption and consequently HBI scores is necessary. The BEVQ-15, a valid and reliable brief food frequency questionnaire, may be a possible rapid method of obtaining HBI scores (administration time of 3–4 min) [25,26,27] as compared to the traditional method of computing HBI scores via dietary recalls/records. Using beverage consumption derived from the BEVQ-15 and estimating energy requirements and subsequently fluid needs, HBI scores may be obtained more efficiently with fewer associated resources.

The ultimate goal of this research is to inform the most rapid, practical, and valid method of determining HBI scores within clinical and community settings. Thus, the objectives of this investigation are to (1) use the BEVQ-15 to obtain HBI scores, henceforth known as the HBI-Q, and explore differences in HBI-Q scores when using estimated energy needs versus reported energy intake, and (2) compare HBI-Q scores versus HBI scores derived using the traditional approach, which includes three 24-h dietary recalls.

## 2. Methods

### 2.1. Subjects and Design

This analysis is a cross-sectional study design which utilized data from two separate investigations. First, baseline participant data (*n* = 301) from the Talking Health Trial (data collected from 2012 to 2014), a randomized controlled trial that studied adults who consumed ≥200 kcal of sugar-sweetened beverages per day [28,29], was included. Participants in Talking Health were recruited in southwest Virginia through a variety of passive and active methods including targeting mailings, flyers, booths at health fairs and free clinics, and word of mouth [30]. The second study was a cross-sectional investigation (data collected in 2016) examining dietary and beverage consumption patterns (*n* = 125). The only eligibility requirement for the second study was age ≥18 years old. No requirements on sugar-sweetened beverage intake were imposed in order to include individuals with different beverage consumption patterns and provide a combined generalizable sample. Participants from the second study were recruited from the surrounding college town of Virginia Tech via traditional methods including listservs, flyers, and word of mouth. Participants from both studies completed the BEVQ-15, three 24-h dietary recalls, and the Godin Leisure Time Exercise Questionnaire [31]. All participants were adults aged ≥18 years old who provided demographic information and anthropometric measurements of height, measured in meters without shoes using a stadiometer; and weight, measured in light clothing without shoes, to the nearest 0.1 kg using a digital scale (model 310GS; Tanita, Tokyo, Japan); and BMI was calculated. Both studies were conducted according to the guidelines laid out in the Declaration of Helsinki, and all procedures involving human subjects were approved by the Virginia Tech Institutional Review Board. Participants provided written informed consent before enrollment.

### 2.2. The Healthy Beverage Index (HBI)

The HBI is a measure of overall beverage intake quality, which is assessed through ten components, including water, sugar-sweetened beverages, total beverage energy, and total fluid requirements [24]. The development and validation of the HBI is described elsewhere [24]. Briefly, HBI scores range from 0 and 100, with higher scores indicating healthier beverage patterns. Individual components and possible scores are described in Table 1. The HBI considers beverage patterns in the context of energy consumption, thus, either reported energy intake or estimated energy needs are necessary for determining fluid needs (i.e., one mL/kcal fluid requirement) [32]. However, as currently validated, the HBI has only been used with reported energy intake. For this investigation, HBI scores were derived using (1) HBI-Q and (2) reported average beverage consumption and total energy intake from three 24-h dietary recalls.

### 2.3. The HBI-Q

The BEVQ-15 measures self-reported habitual beverage consumption (including sugar-sweetened beverages and total beverage fluid ounces, mL, and calories) among 15 beverage categories over the past month [25,26,27]. To investigate the potential impact of using estimated energy needs vs. reported energy intake on HBI-Q scores, HBI scores were determined and assessed using both methods. Estimated energy and fluid requirements were calculated using the Mifflin–St Jeor equation [33] and multiplied by a physical activity factor. To obtain resting energy expenditure from the Mifflin–St Jeor equations, height (cm), weight (kg), age, and sex are needed [33]. Activity factors, determined by the Dietary Reference Intakes for Energy [34], were assigned to the corresponding degree of meeting physical activity recommendations as determined by the Godin Questionnaire, which assesses minutes of reported mild, moderate, and strenuous exercise [31] (Table 2). Reported energy intake was derived from the dietary recalls as stated below. Participants with incomplete BEVQ-15 responses were excluded from the analyses.

### 2.4. HBI via Dietary Recalls

Three 24-h dietary intake recalls were collected for each participant. For Talking Health, the first recall was collected in-person and the two remaining recalls were completed via unannounced telephone calls within a two-week period. The BEVQ-15 and the Godin were also collected on the same day as the first recall. For the second study, the first and third recalls were collected in-person and the second recall was completed via an unannounced telephone call. The multiple pass method was used to collect the recalls, which were completed by trained research technicians who were supervised by a doctoral-level registered dietitian nutritionist [35]. The dietary intake recalls were analyzed using Nutrition Data System for Research (NDS-R) 2011 nutrition analysis software (Nutrition Coordinating Center, University of Minnesota, Minneapolis, MN, USA ). Total daily energy intake was calculated as an average of the three dietary recalls, and average daily beverage consumption was extracted through the NDS-R food group output files.

### 2.5. Statistical Analysis

Statistical analyses were performed using IBM SPSS statistical analysis software (v. 24 for Windows, 2016, SPSS Inc., Chicago, IL, USA). Descriptive statistics, mean (standard deviation) and frequencies, are reported for participant demographic characteristics. Paired-sample *t*-tests and Pearson’s correlations (*r*) were used to assess for differences and associations between (1) HBI-Q scores using estimated energy needs vs. reported energy intake, and (2) HBI scores via the HBI-Q vs. dietary recalls. A Bonferroni correction (11 variables) was applied to set the significance level at *p* ≤ 0.0045. A Bland–Altman analysis was also used to assess the agreement between HBI scores via the HBI-Q vs. dietary recalls. HBI scores were log-transformed due to non-normal data distribution for the Bland–Altman plots.

## 3. Results

### 3.1. Demographics

Twenty-two participants from both studies did not have complete BEVQ-15 data and were excluded from the analyses. The included participants (*n* = 404), combined from both investigations, were primarily female (74%) and Caucasian (87%). The mean reported age was 40 ± 14.5 years old (range 18–86) and the majority of participants were considered overweight or obese (68%), with an average BMI of 31.0 ± 8.9 kg/m^2^ (range 16.1–71.7). Most participants were high school graduates with some college (47%) or college graduates (46%). The remaining participants had less than a high school diploma (7%). Participant completion of all three 24-h dietary recalls was 82% (*n* = 331), with 11% having two days (*n* = 46), and 7% with one day of reported intake (*n* = 27).

### 3.2. Comparison of Estimated Energy vs. Reported Energy HBI-Q Methods

The impact of using the HBI-Q with estimated energy needs vs. reported energy intake was assessed. No significant differences between individual HBI component scores were found, with the exception of the total fluid requirement score (*p* ≤ 0.001); however, the mean difference was minimal (i.e., 0.5 points on a scale of 0–20). The total HBI score between the two methods was not significantly different with a minimal difference of 0.5 on a scale of 0–100 (63.7 for estimated needs vs. 63.2 for actual energy intake). Furthermore, the correlation for total HBI scores between these two methods was significant (*r* = 0.96, *p* ≤ 0.001) (Table 3).

### 3.3. Comparison of HBI Scores via the HBI-Q and Dietary Recalls

**Figure 1:**
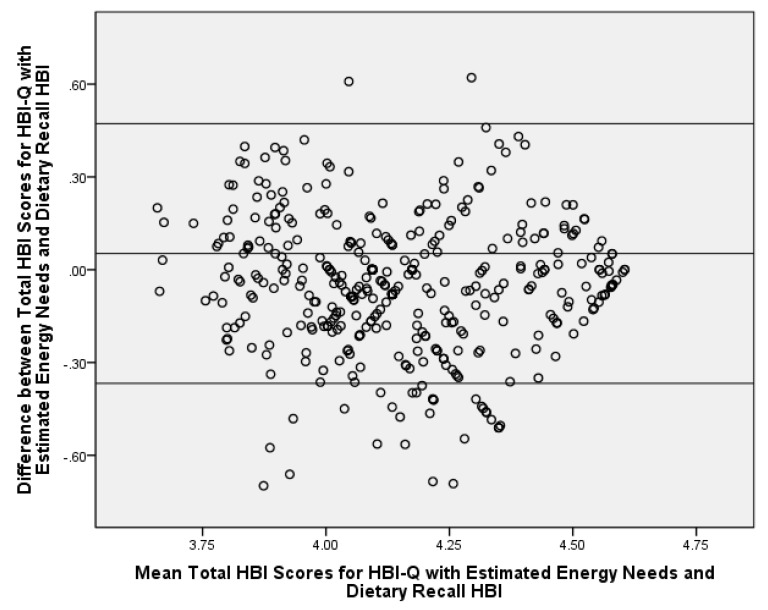
Bland–Altman Analysis of the HBI-Q with Estimated Energy Needs vs. Dietary Recall Healthy Beverage Index (HBI) Scores for Total HBI Scores; Adults (*n* = 404) living in Southwest Virginia, United States, 2012–2016. All values are log-transformed. The center line represents the mean difference and the upper and lower lines indicate the mean ± 1.96 × standard deviation. HBI-Q, a method to rapidly determine Healthy Beverage Index scores.

Using estimated energy needs as a proxy for reported energy intake did not considerably influence calculated HBI-Q scores. Since this is a more practical and rapid approach to assess energy, the next step was to compare HBI-Q scores using estimated energy needs vs. HBI scores via intake from the dietary recalls (Table 4). Several significant differences were found between the HBI-Q and dietary recall HBI scores; however, the mean differences were minimal for individual HBI component scores (differences ranged 0.3–1.2/5 points and 0.9–2.1/20 points). A slightly larger absolute mean difference of 3.6/100 points for the total HBI score was found. The dietary recall method of obtaining HBI scores produced healthier HBI component scores for all of the significantly different HBI scores, with the exception of the total fluid requirement component and the total HBI score, in which the HBI-Q produced higher scores. A significant correlation was demonstrated between the two methods for total HBI scores, (*r* = 0.63, *p* ≤ 0.001). Bland–Altman analysis revealed 92% agreement between the HBI-Q and dietary recall HBI scores for total HBI scores (Figure 1).

## 4. Discussion

This study sought to determine the ability of the BEVQ-15 to rapidly assess HBI scores, and the results demonstrated that the HBI-Q, with estimated energy needs, can rapidly and accurately calculate HBI scores. Importantly, the comparison of HBI scores derived from these two methods reflects the differences between habitual versus recent beverage consumption patterns and the impact that different methodologies may have on reported overall beverage quality scores.

The mean significant difference between many of the components may be explained by the habitual nature of the HBI-Q assessment method (i.e., intake over the past month) versus the recent nature of the 24-h dietary recall HBI assessment method (i.e., intake over three days). Accordingly, it would be unlikely that the scores generated by each method would be identical. Dietary recalls and food-frequency questionnaires both have errors associated with participant reporting [36]. People tend to under-report unhealthy dietary items, such as sugar-sweetened beverages, and may feel pressure to respond to researchers with appropriate answers when completing a dietary recall [37]. This associated error may not be as pronounced with the HBI-Q, as it was self-administered [36]. Furthermore, the HBI-Q is able to measure beverage consumption that occurs less frequently than daily, such as only one to two times per week. If a participant only consumes soda one day per week, there is a possibility that the dietary recalls will not capture this intake. The results of the Bland–Altman plot confirmed these potential differences in the two HBI calculation methods with the agreement being slightly below the standard of 95% agreement [38,39,40]. However, the correlations between total HBI scores for the two methods are consistent with similar reported work, with acceptable correlations ranging from 0.5 to 0.7 [41].

In comparison, the total HBI score from the dietary recalls in this sample (67.3) was similar to HBI scores from National Health and Nutrition Examination Survey (NHANES) 2005–2010 data (*n* = 16,252) (68.2), which are also recall derived scores [24]. Despite the significant difference of 3.6 in total HBI scores between the HBI-Q and the dietary recalls, within the context of health risk, this difference may not be clinically significant. Using the same NHANES data as above, Duffey and Davy found that for each 10-point increase in HBI scores, there was lower risks of hypertension, high fasting insulin levels, high fasting glucose levels, high low-density lipoprotein cholesterol levels, low high-density lipoprotein cholesterol level (women only), and high C-reactive protein level (men only) [24]. It is important to note that HBI scores for this investigation were not examined for possible health risk associations, thus future research with the HBI-Q should be conducted to assess this area.

Of the six significant differences between the HBI-Q and recall methods, only one HBI component, total fluid requirement, was considered less healthy when using the dietary recall method. All scores were improved when using the dietary recall method with the exception of the total fluid requirement component. A possible explanation is that participants reported less consumption of unhealthy beverages (i.e., whole milk, sugar-sweetened beverages) on the recalls, which were researcher-administrated, and consequently less total fluids. Conversely, with the self-administered HBI-Q method, beverage intake of these unhealthy beverages and total fluid consumption was greater. Thus, reported beverage consumption from the HBI-Q may more accurately represent true beverage consumption patterns as compared to dietary recalls.

We acknowledge that a limitation of this analysis is the reliance on self-reported data, which are subject to reporting error and participant bias. However, validated measures of dietary intake were used, and dietary intake data were collected by trained research personnel who were supervised by PhD-level registered dietitian nutritionists. Furthermore, intake was analyzed with state-of-the-art dietary analysis software, NDS-R. Additionally, estimated energy needs must be calculated in order to determine HBI scores using the HBI-Q. Estimated energy need equations can be quickly determined with minimal demographic information (i.e., age, sex, height, and weight); however, determining energy expenditure from physical activity using the Godin Questionnaire may take approximately 5–10 additional min. Due to this reliance on estimated measures for physical activity, these results should be viewed as preliminary and specific to this population. Future work should employ additional direct measures to estimated energy needs, such as physical activity monitors or indirect calorimetry. Despite these limitations, this method is substantially less time-consuming than completing multiple 24-h dietary recalls, and may provide a better estimate of true fluid requirements without the bias of under-reporting caloric intake.

## 5. Conclusions

In conclusion, the HBI-Q can be used to rapidly assess HBI scores in adults, thereby increasing the utility of the HBI by decreasing the time and resources required. This rapid measure of HBI scores will allow researchers and practitioners to efficiently determine the beverage health of patients and provide targeted feedback for improvement. Scoring instructions are available upon request from the corresponding author. Future work should determine cardio-metabolic risks associated with certain HBI score ranges and develop a HBI for use in children.

## Figures and Tables

**Table 1:** Healthy Beverage Index (HBI) components ^a^.

Healthy Beverage Index Components	Description	Points
**Water**	Water comprises ≥20% of fluid requirements	15
	No water consumption	0
	Water is >0% but <20% of fluid requirements	Proportional score between 0 and 15
**Coffee and Tea**	Unsweetened coffee and tea comprise 0–40% of fluid requirements	5
**Low-fat Milk**	≤1.5% fat milk comprises 0–16% of fluid requirements	5
**Diet Drinks**	Artificially sweetened beverages comprise 0–16% of fluid requirements	5
**100% Fruit Juice**	100% fruit juice comprises 0–8% of fluid requirements	5
**Alcohol**	0–1 drinks for women, 0–2 drinks for men	5
**Full-fat Milk**	0% of fluid requirements come from 2% fat or full-fat milk	5
**Sugar-sweetened Beverages**	Sugar-sweetened beverages are 0–8% of fluid requirements	15
**Total Beverage Energy**	Energy from beverages ≤10% of total energy	20
	Energy from beverages ≥15% of total energy	0
	Energy from beverages is >10% but <15% of total energy	Proportional score between 0 and 20
**Met Fluid Requirements ^b^**	Amount of beverages (mL) consumed was ≥ fluid requirements	20
	Amount of beverages (mL) consumed was < fluid requirements	Proportional score between 0 and 20

^a^ The Healthy Beverage Index was previously developed and validated by Duffey and Davy [24]; ^b^ Fluid requirements based on 1 mL/kcal consumed [32].

**Table 2:** Estimating Activity Factors using the Godin Physical Activity Questionnaire ^a^.

Moderate to Vigorous Physical Activity Category ^b^	Strengthening Exercise Category ^c^	Corresponding Activity Factor ^d^
Sedentary		
1	1	1.0
1	2	1.0
1	3	1.0
Does not meet recommendations, but not sedentary		
2	1	1.1
2	2	1.1
2	3	1.1
Meets physical activity recommendations but not strengthening exercise recommendations		
3	1	1.3
3	2	1.3
Meets recommendations for all categories		
3	3	1.5

^a^ Adapted from the Godin Leisure-Time Physical Activity Questionnaire [31]; ^b^ Physical activity category criteria: 1 = Sedentary, ≤9 min of physical activity per week; 2 = Does not meet recommendations but not sedentary, 10–149 min of physical activity per week; 3 = Meets recommendations, ≥150 min of physical activity per week; ^c^ Strengthening exercise category criteria: 1 = Sedentary, 0 times per week of strengthening exercise; 2 = Does not meet recommendations but not sedentary, 1 time per week of strengthening exercise 10–149 min; 3 = Meets recommendations; ≥2 times per week of strengthening exercise; ^d^ Adapted from Dietary Reference Intakes for Energy [34].

**Table 3:** Comparison of Healthy Beverage Index (HBI) scores via the HBI-Q using estimated energy needs vs. reported energy intake (*n* = 404).

Healthy Beverage Index Components (Possible Score)	Comparison of HBI-Q Scores Using Estimated Energy Needs vs. Reported Energy Intake		
	HBI-Q Scores with Estimated Energy Needs ^a^	HBI-Q Scores with Reported Energy Intake ^a^	Mean Difference ^b^
Water (0–15)	12.2 (4.7)	12.1 (4.8)	0.1 (0.1)
Coffee & Tea (0–5)	4.7 (1.1)	4.8 (1.0)	−0.1 (0.04)
Low Fat Milk (Skim or 1%) (0–5)	4.5 (1.6)	4.4 (1.6)	0.1 (0.05)
Diet Drinks (0–5)	4.2 (1.8)	4.2 (1.9)	0.04 (0.05)
100% Fruit Juice (0–5)	4.3 (1.8)	4.2 (1.8)	0.04 (0.1)
Alcohol (0–5)	4.6 (1.4)	4.6 (1.4)	0.0 (0.0)
Whole Fat Milk (≥2%) (0–5)	3.3 (2.4)	3.3 (2.4)	0.0 (0.0)
Sugar-Sweetened Beverages (0–15)	2.6 (5.7)	2.8 (5.9)	−0.2 (0.1)
Total Beverage Energy (0–20)	5.2 (8.2)	5.1 (8.3)	0.2 (0.2)
Met Total Fluid Requirement (0–20)	18.1 (3.4)	17.6 (3.8)	0.5 (0.1) ***
Total HBI Score (0–100)	63.7 (15.8)	63.2 (16.4)	0.5 (0.2)

*** *p* ≤ 0.001, *P* value set at ≤0.0045 based on the Bonferroni Test. ^a^ Reported values are mean (standard deviation); ^b^ Reported values are mean differences (standard error). Mean differences according to paired sample *t*-tests; slight differences may be noted from the preceding columns due to rounding. HBI-Q, a method to rapidly determine Healthy Beverage Index scores.

**Table 4:** Comparison of Healthy Beverage Index (HBI) scores from the HBI-Q and dietary recalls (*n* = 404).

Healthy Beverage Index Components (Possible Score)	Comparison of HBI Scores Derived from the HBI-Q vs. Dietary Recalls		
	HBI-Q Scores with Estimated Energy Needs ^a^	Dietary Recall HBI Scores ^a^	Mean Difference ^b^
Water (0–15)	12.2 (4.7)	11.9 (5.4)	0.4 (0.2)
Coffee & Tea (0–5)	4.7 (1.1)	4.7 (1.2)	0.04 (0.1)
Low Fat Milk (Skim or 1%) (0–5)	4.5 (1.6)	4.7 (1.1)	–0.3 (0.1) ***
Diet Drinks (0–5)	4.2 (1.8)	4.4 (1.6)	–0.2 (0.1)
100% Fruit Juice (0–5)	4.3 (1.8)	4.8 (1.1)	−0.5 (0.1) ***
Alcohol (0–5)	4.6 (1.4)	4.7 (1.2)	−0.1 (0.1)
Whole Fat Milk (≥2%) (0–5)	3.3 (2.4)	4.5 (1.5)	−1.2 (0.1) ***
Sugar-Sweetened Beverages (0–15)	2.6 (5.7)	3.2 (6.2)	−0.6 (0.3)
Total Beverage Energy (0–20)	5.2 (8.2)	7.3 (8.9)	−2.1 (0.4) ***
Met Total Fluid Requirement (0–20)	18.1 (3.4)	17.1 (4.1)	0.9 (0.2) ***
Total HBI Score (0–100)	63.7 (15.8)	67.3 (16.8)	3.6 (0.7) ***

*** *p* ≤ 0.001, *p* value set at ≤0.0045 based on the Bonferroni Test. ^a^ Reported values are mean (standard deviation); ^b^ Reported values are mean differences (standard error). Mean differences according to paired sample *t*-tests; slight differences may be noted from the preceding columns due to rounding.
